# A Machine Vision‐Guided Microphysiological Platform With Automated Microfluidics Enables Longitudinal Biomarker Monitoring and Emulation of Translationally Relevant Exposure Scenarios

**DOI:** 10.1002/advs.76256

**Published:** 2026-06-22

**Authors:** Jibbe Keulen, Yi Zhong, Laura Dangel, Sonia Youhanna, Reza Zandi Shafagh, Jinhye Ryu, Domnica‐Gabriela Gurau, Mathias Haag, Thomas E. Mürdter, Yingxin Liang, Iro Beligiani, Nicole Ziegler, Sabine Willems, Nayere Taebnia, Wouter van der Wijngaart, Volker M. Lauschke

**Affiliations:** ^1^ Dr. Margarete Fischer‐Bosch Institute of Clinical Pharmacology Stuttgart Germany; ^2^ University of Tübingen Tübingen Germany; ^3^ Department of Physiology and Pharmacology and Center for Molecular Medicine Karolinska Institutet and University Hospital Stockholm Sweden; ^4^ Division of Micro‐ and Nanosystems KTH Royal Institute of Technology Stockholm Sweden; ^5^ Pharmaceutical Informatics Institute College of Pharmaceutical Sciences Zhejiang University Hangzhou China; ^6^ Fraunhofer Institute for Translational Medicine and Pharmacology (ITMP) Frankfurt am Main Germany; ^7^ Department of Pharmacy the Second Xiangya Hospital Central South University Changsha China

**Keywords:** microfluidics, microphysiological systems, organ‐on‐a‐chip, organotypic culture, translational toxicology

## Abstract

Low success rates in clinical drug development can be largely attributed to the poor predictive power of existing preclinical models. Microphysiological systems (MPS) have greatly advanced in vitro modeling; however, current platforms do not adequately support long‐term sampling and often fail to recapitulate nutrient and drug exposure dynamics. To address these limitations, we established a machine vision‐guided MPS with real‐time fluidic control that enables fully automated periodic sampling with high temporal resolution, media replenishment, and programmable dosing, allowing for the simulation of dynamic nutritional or pharmacological exposure scenarios. We showcase the system's capability by emulating physiological insulin profiles and repeated‐dose pharmacokinetic exposures over multiple weeks. Furthermore, pharmacokinetically accurate acetaminophen exposure in 3D primary human liver spheroids mimicking an acute overdose rapidly induced liver toxicity, as evidenced by aminotransferase release, cytokine secretion and a drop in cellular ATP. In contrast, dose‐equivalent constant exposure patterns did not elicit detectable hepatotoxicity. Mechanistically, targeted proteomics of sampled supernatants and Cell Painting revealed that toxicity was paralleled by disrupted lipid homeostasis, loss of tight junctions and extracellular matrix remodeling. These results demonstrate the robustness and versatility of the machine vision‐guided automated microphysiological platform and underscore the importance of incorporating drug exposure dynamics for mechanistic toxicology.

## Graphical abstract

A pneumatically actuated multi‐tissue microphysiological system is integrated with AI‐based machine vision and automatic sampling and replenishment systems. The platform allows for the emulation of translationally relevant long‐term pharmacokinetic exposure scenarios for multiple weeks while enabling longitudinal monitoring of response biomarkers. Acetaminophen toxicity emerges under dynamic exposure, but not under dose‐equivalent constant exposure, revealing how exposure profiles shape mechanistic hepatotoxicity.

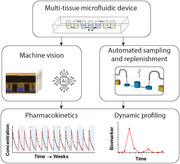

## 1 Introduction

Over the past decade, the cost of drug development has skyrocketed from $161 million to $4.4 billion per drug [1]. Among major pharmaceutical companies, the likelihood of approval for drug candidates entering clinical trials is only 14.3% [2], and success rates are even lower (4.9%) when considering all clinical drug development programs [3]. Given that the cost of failed programs accounts for approximately 2/3 of the total cost of drug development [4], these numbers demonstrate that the poor translation of promising preclinical findings into successful clinical programs constitutes a main driver of pharma R&D spending. A major contributor to these failures is the lack of preclinical systems that can reproduce the dynamic physiological environments to which human tissues are exposed, or that can monitor tissue function continuously in real time.

This translational gap stems in part from the limited predictive capacity of current preclinical models. Animal models exhibit fundamental limitations due to interspecies differences that lead to the identification of ineffective molecular targets, large variation in drug‐dose responses, and, at times, poor resemblance to human pharmacokinetics and pharmacodynamics [5]. Consequently, among 76 highly cited animal studies published in top journals, the outcomes of only 37% were confirmed in human studies [6]. Likewise, in a systematic evaluation of six interventions with well‐established clinical benefits across 221 human studies, only half showed comparable effects in animal models, whereas the others did not [7]. The predictive power of safety studies has been similarly poor. Preclinical animal testing identified just 19% of 93 serious post‐marketing adverse reactions associated with 43 small‐molecule drugs [8], and a lack of toxicity in animals was only a weak indicator of human safety [9]. Inversely, increased confidence in preclinical safety data correlated strongly with a decrease in clinical toxicity events [10].

Organotypic culture formats, such as spheroids or organoids, enable the long‐term culture of phenotypically relevant human cells. A multitude of culture methods and protocols have been developed for an ever‐growing number of human tissues, including liver, intestine, kidney, heart, brain, and muscle [11, 12]. In contrast to conventional 2D cultures, which often deteriorate within days, some of these 3D models allow for stable culture for weeks to months, enabling the study of long‐term human drug absorption, distribution, metabolism, and excretion (ADME) as well as chronic toxicity. Cognizant of these results, regulatory change, formalized in the FDA Modernization Act, now allows for the use of in vitro methods to be included as part of a safety and efficacy package. However, even advanced 3D systems typically lack automated, high‐temporal‐resolution sampling and the ability to control or monitor exposure conditions continuously, meaning that the biological sophistication of these tissues is not yet matched by equally advanced fluidic or control architectures. This is particularly important given that pharmaceutical companies use advanced culture models predominantly for mechanistic studies [13]. Thus, while generally seen favorably [14], there remain concerns about whether the current in vitro models are ready to replace animal studies in regulatory packages [15, 16, 17].

Microphysiological systems (MPS) in which human cells are cultured within microfluidic devices aspire to become a game changer that combines the human phenotypic relevance of organotypic cultures with precise control of environmental dynamics [18, 19]. However, the vast majority of systems expose the cultured cells to constant concentrations throughout the course of an experiment. This misrepresents in vivo conditions, where both nutritional and pharmacological stimuli follow distinct temporal patterns. These differences in temporal dynamics can have profound biological implications. For instance, postprandially liver cells adjust glycolysis and glycogenesis to changes in insulin concentrations rather than to absolute insulin concentrations [20]. Similarly, pharmaceutical compounds undergo continuous concentration changes in vivo due to absorption, distribution, metabolism, and excretion (ADME) processes, where circulating levels typically increase rapidly following administration and gradually decline thereafter as they are cleared from the body. Furthermore, existing MPS architectures seldom incorporate real‐time feedback control of flow or volume, rarely support precise microliter‐scale sampling or replenishment, and often rely on materials such as PDMS that extensively absorb small molecules. Moreover, current systems cannot easily execute programmable exposure profiles from multiple reservoirs nor store frequent samples in an automated, scalable manner, leaving core technological gaps in the fluidic control of dynamic experiments.

To address these limitations, we developed a microfluidic platform with pneumatic actuation that enables automated longitudinal sampling with high temporal resolution and allows for the emulation of physiologically and pharmacologically relevant exposure dynamics. By interfacing a fully automated pressure controller and valve system with a machine vision‐based real‐time fluidic controller, the setup allows periodic sampling and medium replenishment without manual intervention and programmable dynamic dosing to simulate pharmacokinetic profiles and repeated nutritional challenges. We demonstrate that dynamic exposure to acetaminophen (APAP) overdose regimens caused acute hepatocellular toxicity, whereas continuous exposure to equivalent levels did not result in liver injury. Targeted proteomics profiling of cellular secretomes revealed signatures of injury from both hepatocytes and non‐parenchymal liver cells. In summary, we present a microfluidic platform for the dynamic long‐term culture of 3D primary human microtissues that incorporates transformer‐based machine‐vision algorithms for real‐time monitoring of chamber volumes, while air‐segmented in‐line sample storage supports scalable, high‐frequency sampling without manual handling. Built from low‐sorption materials, the system is optimized for accurate pharmacological studies and allows for the faithful recapitulation of complex exposure regimens.

## 2 Materials and Methods

### 2.1 Chip Fabrication and Assembly

Microfluidic chips were produced by micro reaction injection molding (µRIM) using off‐stoichiometric thiol‐ene‐epoxy (OSTE+) polymers (OSTEMER‐322; Mercene Labs, Sweden) as described previously [21]. Polymethyl methacrylate (PMMA) layers were produced using micromilling, and the needles connected to the pressure controller were inserted into the L‐shaped channel and sealed off using Loctite precision glue (Loctite, Germany). Polydimethylsiloxane (PDMS) layers were produced by casting in aluminum molds and subsequent degassing. Devices were assembled by bonding the OSTE+ layers and aligning them with the PDMS and PMMA parts, followed by insertion of the assembled device into a custom‐made aluminum clamping frame for tight sealing.

### 2.2 Sampling System

The sampling system was based on an OB1 MK3+ pressure controller and a MUX distribution/injection valve system (Elveflow, France). The MUX valve system was linked to the OB1 pressure port (‐1000/6000 mbar pressure range) through 4 mm OD coil tubing (Sigma‐Aldrich, USA), which was subsequently attached to 0.8 mm ID/1.6 mm OD polytetrafluoroethylene (PTFE) tubing (Merck, Germany) through microfluidic connectors. A 20 cm PEEK resistance tubing with an inner diameter (ID) of 65 µm was added. The MUX valve system was connected to the sampling and replenishment ports, sampling storage ports, and media reservoirs using 0.8 mm ID/1.6 mm OD PTFE tubing (Merck, Germany). The sampling and replenishment ports were in turn connected to the microfluidic device through stainless steel microfluidic fittings that were pushed through a rubber septum (Sigma‐Aldrich, USA) that was inserted into the tissue chamber opening in the top PMMA layer. The stainless‐steel microfluidic fittings used for sampling and replenishment were positioned at a height that ensured submersion only upon overpressure of the pneumatically actuated device, that is, when the medium was pumped out from the central chamber and into the peripheral satellite chambers. The system was operated using ESI software (Elveflow, France) and experiments controlled by custom scripts based on the SDK Python libraries (Elveflow, France).

### 2.3 Cell‐Free Sampling and Replenishment Experiments

To visualize mixing, cell‐free experiments were performed using blue dye as a proxy. The brilliant blue dye (Dr. Oetker, Germany) was diluted to concentrations of 0.005–0.025 mg/ml. Dye and deionized water, respectively, were primed into replenishment tubing by using negative pressure to draw in the medium into the appropriate tubing until it reached the MUX valve system, at which point the excess medium was pumped into the waste port.

### 2.4 Machine Vision

To establish the feedback system, a long working‐distance USB‐microscope (DinoLite) was placed parallel to the flow plane facing the front of the chip. An image was taken from the camera every 1–3 s and analyzed using the SegFormer semantic segmentation framework [22]. After segmentation, the distance from the top of the fluid level to the base of the chamber was measured and exported. These distances were used to operate the pumping process in the microfluidic chambers by setting lower and upper thresholds at which the pressure would be reversed, sequentially pumping fluid from the central chamber to the tissue chambers and vice versa. Upper and lower parameters were selected based on the fluid displacement necessary to facilitate effective mixing and molecular transport between the tissue chambers. For volumetric determination, the distances in each chamber were used to calculate the volumes in each chamber and subsequently the volume in the entire chip.

### 2.5 Experimental Preparation

Before each experiment, all tubing and connectors were flushed with ethanol and subsequently dried to ensure complete ethanol evaporation. All tubing exposed to samples was coated with a 1% BSA solution (Sigma, USA) to prevent non‐specific adsorption. The components of the microfluidic chips were cleaned by immersion in ethanol overnight and subsequent irradiation with UV light for 15 min. Before use, the microfluidic chips were coated with BIOFLOAT FLEX coating solution (faCellitate, Germany). After coating, the chips were washed with culture medium to remove any remnants of the coating solution, and the microfluidic channels of the chip were primed by pipetting medium up and down in close proximity to the channel entrance. Replenishment medium was primed by using negative pressure to draw in the medium into the appropriate tubing until it reached the MUX valve system, at which point the excess medium was pumped into the waste port. It was crucial that the medium in the replenishment tubing was in direct contact with the valve to ensure precision of the replenishment volumes.

### 2.6 Liver Spheroid Culture

Primary human liver spheroids were cultured as described previously [23]. In brief, primary human hepatocytes (PHH) and non‐parenchymal cells (NPCs) were thawed and seeded in 96‐well or 384‐well ultra‐low attachment (ULA) plates (Biofloat, faCellitate, Germany) in liver culture medium (Williams’ E medium supplemented with 10 µg/ml insulin, 2 mM L‐glutamine, 100 U/ml penicillin, 100 µg/ml streptomycin, 5.5 µg/ml transferrin, 6.7 ng/ml sodium selenite, 100 nM dexamethasone) supplemented with 10% fetal bovine serum (FBS). Demographic and medical information about the donors is provided in Table S1. In 96‐well plates, we seeded 1500 PHH, whereas 500 PHH were seeded per spheroid in 384‐well plates. NPCs were added to the spheroids at the time of seeding at a PHH:NPC stoichiometry of 4:1. After 5–6 days, when the spheroids were sufficiently compact, FBS was phased out. The culture of primary human cells was approved by the national governing body (Etikprövningsmyndigheten, Sweden) under permit number 2024‐05808‐01.

### 2.7 Pharmacokinetic Experiments

PHH spheroids were transferred into the satellite chambers of the microfluidic device at a density of 25 spheroids per chamber in liver culture medium. Subsequently, the devices were assembled and connected to the appropriate ports for pneumatic actuation, sampling and replenishment. The USB‐microscope was aligned to the three central chambers of the devices. For dynamic exposure, the sampling and replenishment protocol was programmed to emulate in vivo APAP exposure patterns, that is, increasing concentrations up to maximum peak plasma concentration (c_max_) at T_max_ and decreasing thereafter with the elimination half‐life (T_1/2_). Values for c_max_ (2.5 mM and 5 mM), T_max_ (1 h) and T_1/2_ (2 h) are based on patient data from human APAP overdoses [24]. Constant APAP concentrations with the same area under the exposure curve (AUC_exposure_) were used as a comparator. APAP concentrations were measured by mass spectrometry as previously reported [25]. The protocol was otherwise identical to the dynamic exposure profile in volumes sampled and replenished. The total run time of both exposure programs was 24 h.

### 2.8 Cell Viability

Viability of liver spheroids was assessed using the CellTiter‐Glo cell viability assay kit (Promega, USA). Briefly, individual spheroids were transferred in 50 µl media to white opaque 96‐well plates (Corning, USA) and equilibrated at room temperature for 30 min. Afterwards, 50 µl of CellTiter‐Glo reagent was added to each well, followed by incubation at room temperature for 10 min on an orbital shaker. The cells were then left for another 2 min for stabilization of the luminescent signal. Luminescence was then measured using a Spectramax ID3 multi‐mode plate reader (Molecular Devices, USA).

### 2.9 Enzyme‐Linked Immunosorbent Assay

Hepatocellular injury was evaluated by quantifying alanine aminotransferase (ALT), and aspartate aminotransferase (AST) release in collected samples. ALT and AST were quantified using human ALT and AST enzyme‐linked immunosorbent assay (ELISA) kits (Abcam, UK) according to the manufacturer's protocols. Briefly, culture medium was collected at the end of the exposure period and centrifuged at 10 000 g for 5 min. Samples were added to the pre‐coated microwell plates along with the provided standard and incubated at room temperature for 2 h. After washing, detection antibodies were applied, followed by incubation with substrate development solution and the stop solution. Absorbance was measured at 450 nm on a Spectramax ID3 multi‐mode plate reader (Molecular Devices, USA).

### 2.10 Cell Painting

Cell Painting Assay was performed using the PhenoVue Cell Painting JUMP Kit (Revvity, Inc., USA) with adaptations to 3D culture and dye selection as follows. PhenoVue 641 mitochondrial stain was added to the live spheroids at a final concentration of 0.5 µM, the cells were centrifuged at 200 g for 1 min and incubated for 3 h at 37°C with 5% CO_2_. Thereafter, spheroids were fixed in 4% methanol‐free paraformaldehyde solution and centrifuged again at 200 g for 1 min. After 40 min of fixation at room temperature in the dark, the spheroids were washed 3× with HBSS buffer. A staining solution containing PhenoVue Fluor 555‐WGA (final conc. 1.5 µg/ml), PhenoVue Fluor 568‐Phalloidin (final conc. 8.25 nM), PhenoVue Hoechst 33342 nuclear stain (final conc. 1 µg/ml), and BODIPY 493/503 (final conc. 0.52 µg/ml; Invitrogen, USA) was prepared in PhenoVue dye diluent A supplemented with 0.1% Triton X‐100 and added to the spheroids. After centrifugation at 200 g for 1 min, the spheroid plate was sealed and incubated at 4°C overnight in the dark. The following day, spheroids were washed 3× with HBSS buffer and centrifuged (200 g, 1 min). A pre‐warmed clearing solution containing 2.5 M fructose and 60% (v/v) glycerol was added, and spheroids were cleared for 3 days at room temperature in the dark prior to imaging. Cell imaging was performed using a 20× air objective on a high‐throughput confocal microscope (Opera Phenix, Revvity).

### 2.11 Quantitative Multivariate Analysis of the Cell Painting Data

After acquiring the Cell Painting images, 3D segmentation was implemented using a two‐step segmentation pipeline combining CellPose [26] and u‐Segment3D [27], followed by morphologic feature extraction with CellProfiler [28] to quantify area, convex area, major and minor axis lengths, extent, solidity, eccentricity, and perimeter for both cell and nuclear compartments on a per‐cell basis. Per‐well summaries were obtained by aggregating single‐cell measurements and combined with self‐supervised DINO v3 [29] embeddings, which were derived by applying a pretrained DINO v3 backbone to preprocessed image tiles and pooling patch‐level representations into a single embedding vector per channel. The resulting tabular dataset of morphologic features and embeddings was used to train a TabPFN [30] classifier to predict compound‐level drug‐induced liver injury (DILI) labels. For the statistical comparison, extracted morphologic features were contrasted between APAP‐treated spheroids and the corresponding isogenic vehicle controls by normalizing APAP feature distributions to the average of DMSO wells. Statistical significance was assessed using heteroscedastic T tests.

### 2.12 Olink Protein Expression

Cell‐culture supernatants were collected under sterile conditions and clarified by centrifugation at 10 000 g for 5 min to remove cells and debris. Samples were immediately aliquoted and stored at −80°C until analysis. Prior to measurement, supernatants were thawed on ice and diluted according to Olink recommendations for the selected Target panels. Proteomic profiling was performed using the Olink proximity extension assay technology Target 96 Inflammation panel following the manufacturer's instructions (Olink Proteomics, Sweden). Briefly, 1 µl of each sample was incubated with matched pairs of oligonucleotide‐labeled antibody probes. Upon binding to their target protein, the probes were brought into proximity, allowing hybridization and extension by DNA polymerase to generate a unique reporter sequence for each protein. The resulting DNA amplicons were quantified using real‐time PCR. Data were processed using Olink Normalized Protein eXpression (NPX) Signature Software. Protein levels were reported as NPX values on a log2 scale.

### 2.13 Statistical Analyses

For microfluidic system characterization, coefficients of variation (CV) and correlation coefficients (R^2^) were calculated using R (2023.12.1). To evaluate differences between groups, we used one‐way ANOVA followed by Tukey's multiple comparison test in Prism (10.6.1, GraphPad Software, USA). The number of independent replicates (n) is indicated in the respective figure legends. P‐values < 0.05 were considered statistically significant. Data are presented as mean ± standard error of the mean (SEM).

## 3 Results

### 3.1 Design, Setup, and Functionality of the Microfluidic Setup

From a technical perspective, we aimed to implement a microfluidic platform with media recirculation that would allow for complex dynamic exposure regimens, longitudinal automatic sampling, and reciprocal mass transfer between different modular compartments, ideally allowing for compartment‐specific perfusion rates. In addition, the system should fulfill biological and pharmacological requirements, including compatibility with long‐term culture of 3D primary human tissue models and minimal absorption of drugs and other molecules. To this end, we used a pneumatically actuated co‐culture platform comprising a central chamber interfaced with the pressure controller and multiple satellite compartments which are connected to the central chamber via microfluidic channels. Introducing air into or retracting air from the central compartment using the pressure controller, that is, by applying over‐ or underpressure, could drive medium flow from the central chamber to the satellite compartments and back, creating a “synthetic heartbeat” that facilitates mass transfer between the chambers. In contrast to stepwise manual medium exchanges, this bidirectional pumping generates smooth, cyclical mixing that is well suited for emulating time‐varying exposure patterns while maintaining a conditioned medium within a closed recirculating circuit. We previously demonstrated that the system can be stably operated for multiple weeks and supports the culture of organotypic human liver spheroids [21]. Furthermore, the device exhibits drastically lower drug absorption compared to devices made from PDMS [31]. The choice of OSTE as bulk material therefore directly addresses the need for MPS platforms that preserve free drug concentrations and avoid sorption artefacts that limit the translational relevance of many PDMS‐based systems.

**FIGURE 1:**
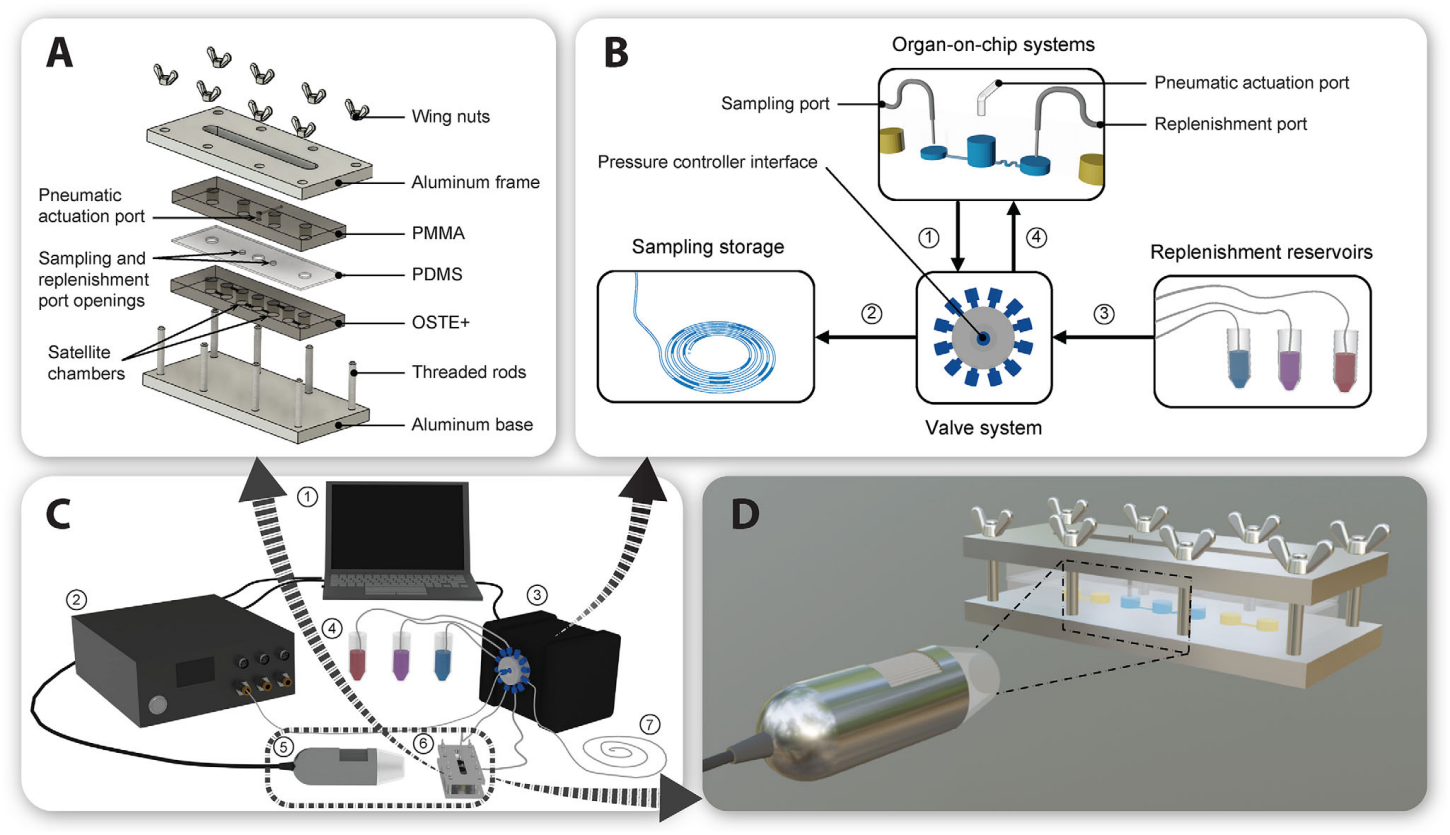
Microfluidic setup and working principles. (A) Schematic overview of the experimental setup, consisting of a laptop computer running custom Python scripts for automated control of all system components (1), a pressure controller capable of applying positive and negative pressure (2), a multiplexed valve manifold (3), reservoirs with different media compositions (4), a USB microscope (5) for real‐time images of the microfluidic chip (6) and tubing for sample storage (7). (B) Exploded view of the microfluidic device assembly, showing the aluminum clamping frame, polymethyl methacrylate (PMMA) top layer connecting to the pressure source, flexible polydimethylsiloxane (PDMS) layer for air‐tight sealing, and the microfluidic device made from off‐stoichiometric thiol‐ene‐epoxy (OSTE). (C) Detailed view of the machine vision setup showing the USB microscope positioned to capture side‐view images of the chip, which are processed using a SegFormer‐based segmentation algorithm on the connected laptop computer to determine fluid levels in real‐time. (D) Schematic representation of valve port connectivity. Of the 12 available ports, two are interfaced with the microfluidic device for sampling and replenishment, one is connected to the pressure controller, one is connected to the storage tubing and the remaining ports can be linked to up to eight different medium reservoirs.

Here, we extended the previously described chip with multiple functional features. Firstly, we introduced sampling and replenishment ports in the microfluidic device, which are located in the satellite chambers intended for the culture of 3D human tissue models (Figure 1A). These ports are connected to a multiplexed valve manifold with 12 ports (Figure 1B). Besides these two ports for sampling and replenishment, one is used for pneumatic actuation, and one is interfaced with the sample storage tubing. Furthermore, the valve manifold is connected to the pressure controller via a central pressure port. This leaves up to eight ports for different media formulations that can be alternated between in a single experiment. The pressure controller and valve system are controlled by a dedicated computer setup to run customized scripts for precise microfluidic control (Figure 1C). Through the pressure controller and valve system, the platform can extract and introduce small media volumes from and into the microfluidic device. The integration of a camera allows for real‐time imaging of fluid levels in the chambers, facilitating machine‐vision based feedback control (Figure 1D).

This setup fulfills three main purposes: firstly, it facilitates stepwise medium replenishment in small increments, that is, fresh medium is added, and waste products are removed in small increments without compromising medium conditioning by the cultured tissue models. Secondly, the platform enables sampling of small volumes of the culture medium with high temporal resolution, which can be used for downstream biomarker evaluation. Thirdly, by tapping into media reservoirs with different compositions, the system can precisely modulate the concentrations of specific compounds or nutrients, which facilitates the emulation of physiologically relevant nutritional or pharmacological exposure dynamics. Accordingly, the integration of sampling and replenishment ports, together with a multi‐reservoir valve manifold, directly responds to the need for scalable, automated longitudinal sampling and for programmable dynamic exposure regimens that cannot be achieved with conventional single‐pass or manually operated closed systems.

### 3.2 Machine Vision‐Guided Feedback for Fluidic Control

**FIGURE 2:**
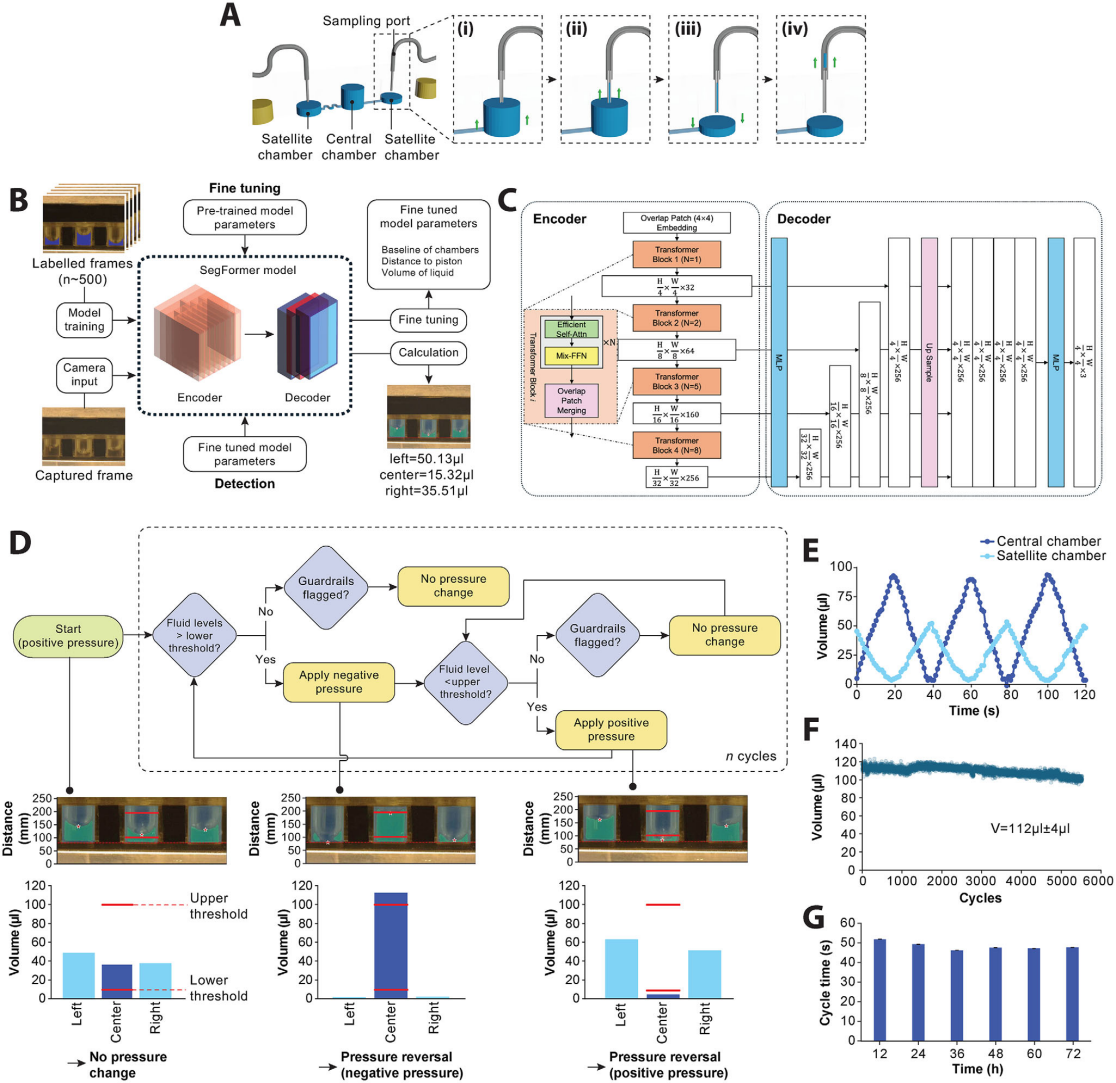
A machine vision‐guided automated sampling and replenishment system. (A) Schematic illustration of the sample extraction process from the microfluidic devices. (i) Fluid is pushed out into the peripheral satellite chambers, immersing the sampling needle. (ii) The desired volume is then extracted by applying negative pressure to the sampling valve. (iii) Afterwards, fluid is retracted into the central chamber, re‐exposing the sampling needle to air. (iv) Subsequently, the sample is drawn out into the sampling tubing and transported to the sampling storage reservoir. (B) A machine vision algorithm based on a SegFormer model architecture is used for real‐time quantification of media volumes in each chamber. Approximately 500 manually labeled frames were used to fine‐tune the encoder–decoder segmentation model. The network was initialized with pre‐trained parameters and optimized using AdamW (learning rate = 1 × 10^−^^6^) for 200 epochs to generate the final fine‐tuned model parameters. The fine‐tuned model was applied to each captured frame to detect key structural features, including the chamber baseline and the piston–chamber interface. Based on these landmarks, the pipeline automatically calculated the piston–chamber distance and converted it to the corresponding liquid volume through geometric computation. (C) Schematic architecture of the SegFormer model used for semantic segmentation. The model consists of a hierarchical Transformer‐based encoder with a mixed feedforward network (FFN) that extracts multi‐scale features and a lightweight multilayer perceptron (MLP)‐based decoder that fuses these features to produce high‐resolution segmentation maps. (D) Flowchart illustrating the feedback algorithm used to control pneumatic actuation. At the start, positive pressure is applied, after which the camera captures and processes a frame every 1 to 3 s. When fluid height in the central chamber reaches the lower threshold, the pressure controller applies negative pressure to draw fluid back into the central chamber. The pressure controller continues to apply negative pressure until the upper threshold in the central chambers is reached, at which point it reverts back to positive pressure. This cycle repeats to maintain continuous bidirectional flow and mixing to ensure efficient communication between the tissue chambers. The lower threshold is defined at a point where the central chamber is nearly empty, and the upper threshold is defined at a point where the peripheral chambers are nearly empty, to ensure efficient mixing of all media and to prevent bubble formation. Guardrail conditions, including prolonged pumping and unusually large fluctuations in volume measurement, are used to detect and mitigate anomalies in pumping behavior. (E) Quantification of fluid volumes in the central chamber and satellite compartments throughout three representative cycles. Each point represents an individual SegFormer measurement. (F) Long‐term stability of the combined volume of all three chambers is plotted over a 3 day period encompassing a total of 5516 pumping cycles. (G) Average cycle duration measured at designated timepoints. Each point represents the average of 10 cycles, and error bars represent the SEM.

The valve system connects to the microfluidic device through two separate ports, one for sampling and one for replenishment. The sampling port is positioned in a way that the medium can only be removed during a phase of the pneumatic actuation cycle in which the medium is pushed out of the central chamber and into the satellite compartments (Figure 2A). This serves as a fail‐safe mechanism to ensure that frequent automated sampling does not deplete the culture volume, addressing the need for high‐temporal‐resolution readouts without compromising long‐term tissue viability in a closed‐loop system. Afterwards, the medium is drawn into the central chamber until the sampling needle is exposed to air, and the sample is subsequently transported through the valve system and into the sample storage tubing. This air segmentation enables the storage of large amounts of samples in the same tubing without the need for direct transfer into storage containers. By enabling air‐segmented sample trains in a single outlet line, the system prevents back‐mixing between samples and overcomes practical scalability limits of manual tube‐based collection and facilitates dense temporal profiling of secreted biomarkers. After the sampling procedure is complete, the sampled volume is replenished by extracting medium from the desired reservoir, which is then transported through the valve system and introduced into the microfluidic chip through the replenishment port.

Long‐term experiments in semi‐closed microfluidic devices require a system that is not affected by evaporation, losses in pumping efficiency, or variations caused by slight pressure differentials or sampling inefficiencies. To achieve this, we implemented a machine vision solution that uses imaging data from a USB‐microscope to adjust the microfluidic program in real‐time. This setup controls pneumatic actuation, assuring that the central chamber is emptied in the positive pressure phase and that not too much medium is withdrawn in the negative pressure phase, thus allowing for a full range of motion independent of the volume inside the chip. Furthermore, the system continuously checks for volumetric changes and, via feedback to the sampling algorithm, automatically compensates for any changes in volume in the subsequent sampling/replenishment cycle. In this way, real‐time volume monitoring and feedback directly address the instability and drift that currently limit the duration and reliability of closed‐loop MPS experiments, enabling autonomous operation over many thousands of cycles without manual recalibration.

To minimize imaging data volume in long‐term experiments, we captured 0.3–1 frames per second (fps) and analyzed the images in real‐time with a SegFormer machine vision algorithm (Figure 2B). The model exhibited a mean intersection over union (IoU) of 0.962 and a mean segmentation accuracy of 0.979. The image was segmented automatically to recognize fluid levels in each compartment. The media volumes in each compartment and in the entire chip were then calculated based on the known feature dimensions (Figure 2C). To reduce model computing resources, we chose the B0 SegFormer model variant with 3.7 million pre‐trained parameters as the base model for fine tuning. The model consists of a hierarchical Transformer‐based encoder with a mixed feedforward network (FFN) and a lightweight multilayer perceptron (MLP)‐based decoder. The input images are first encoded into low‐dimensioned vectors to extract multi‐scale features, which are then decoded to generate native‐resolution segmentation maps for downstream calculation. This real‐time analysis was then used to operate the pneumatic actuation inside the microfluidic system by integrating it into a hysteretic threshold‐based switching algorithm (Figure 2D). This algorithm switched the pressure to positive once the upper threshold was reached, decreasing the volume in the central chamber by pushing fluid into the peripheral chambers. Subsequently, when the lower threshold was reached, the algorithm switched to negative pressure, drawing fluid back into the central chamber. By coupling transformer‐based semantic segmentation with hysteretic threshold control, the platform generates reproducible, well‐defined flow cycles which are required to emulate pharmacokinetic and hormonal exposure patterns with high fidelity.

Long‐term stability of the feedback‐based pumping system was assessed by continuous operation over a three‐day period (Figure 2E). During this period, the system tightly maintained a total volume of 112 ± 4 µl over a total of 5516 pumping cycles (Figure 2F). The feedback system slightly adjusted cycle times from 52 to 48 s, possibly compensating for minor pressure losses within the device (Figure 2G). Combined, these results demonstrate that the machine vision algorithm provides effective feedback control and ensures that the system maintains stable long‐term culture while executing programmable dynamic exposure regimens and dense longitudinal sampling.

### 3.3 Accurate Emulation of Dynamic Exposure Scenarios

**FIGURE 3:**
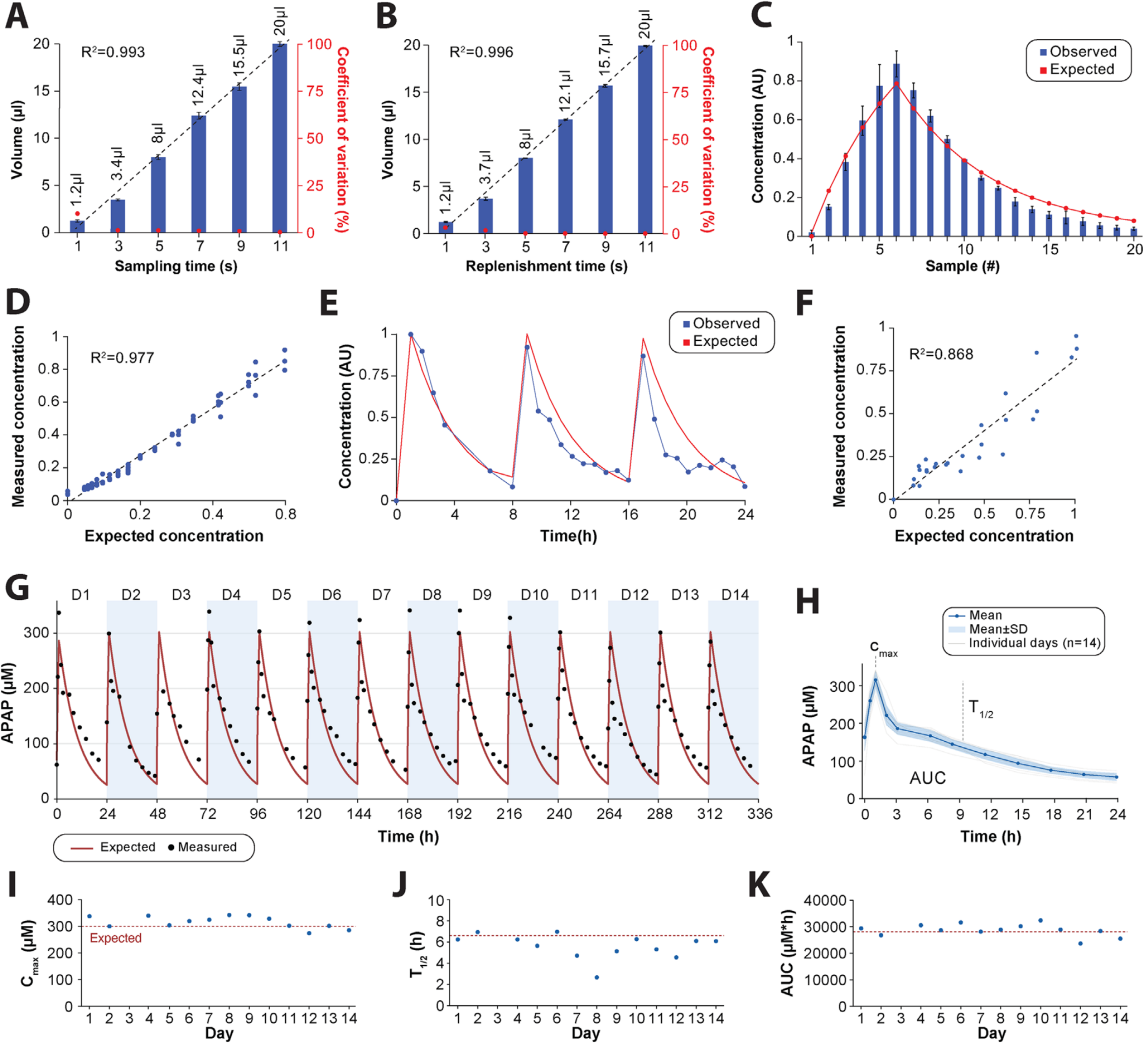
Benchmarking of sampling precision for the emulation of dynamic exposure programs. (A) Sampled volume as a function of time during which negative pressure is applied to the sampling valve (left *y*‐axis). The dashed line indicates the linear correlation between sampling time and sampled volume. Data are shown as an average of 10 measurements. Red dots indicate the variance of the sampled volume expressed as the coefficient of variation (right *y*‐axis). Error bars represent SEM. (B) Replenished volume is shown as a function of time during which positive pressure is applied (left *y*‐axis). The dashed line indicates the linear correlation between time and volume introduced into the microfluidic device. Data are shown as an average of 10 measurements. Red dots indicate the variance of the replenished volume expressed as the coefficient of variation (right *y*‐axis). Error bars represent SEM. (C) Emulation of a prototypic pharmacokinetic exposure profile. The sampling and replenishment system was used to generate a rapid increase and subsequent decrease in concentration in 20 sampling steps. The red line shows the theoretical concentrations while the blue bars represent the actual measurements. Results are shown for *n* = 3 experiments; error bars represent SEM. (D) Theoretical and measured concentrations of the pharmacokinetic profile in panel C show very high concordance (R^2^ = 0.977). (E) Emulation of a prototypic hormonal exposure profile. The system was used to mimic repeated daily insulin exposure patterns over a 24 h period. The red line shows the theoretical concentrations while the blue curve represents the actual measurements. (F) Theoretical and measured concentrations of the hormonal exposure profile in panel E are highly correlated (R^2^ = 0.868). (G) Emulation of a translationally relevant long‐term exposure scenario with single daily exposure. Acetaminophen (APAP) was used as an exemplary drug and concentrations were measured at 12 timepoints each day (5 min, 30 min, 1 h, 2 h, 3 h, 6 h, 8 h, 11 h, 14 h, 17 h, 20 h and 24 h) by mass spectrometry to quantify in‐chip concentration profiles. The red line shows the theoretical concentrations while the black dots represent the actual measurements. (H) Daily concentration profiles are stable for at least 14 days. Average in‐chip concentrations are shown as the blue line, ± one SD intervals are shown in light blue and individual daily traces are shown in grey. (I‐K) Measured pharmacokinetic parameters pivot around the expected values for c_max_ (I), T_1/2_ (J) and the area under the exposure curve (AUC; K) without an obvious drift over time.

Next, we characterized the precision of our sampling system, by extracting samples at a constant pressure over different time intervals (Figure 3A). Sample volumes ranging from 1.5 to 20 µl could be extracted within reasonable timeframes of <11 s, resulting in cycling times of approximately 50 s for a full pumping cycle. Importantly, sampling times correlated excellently with sampled volumes (R^2^ = 0.993). The coefficient of variation (CV) ranged from <1% for sample volumes of 4 µl to 6.9% for 1.5 µl samples, demonstrating excellent reproducibility. Replenishment was even more accurate with CVs <2% across the entire sampling interval (Figure 3B).

Given the high technical accuracy of the sampling and replenishment system, we next simulated dynamic exposure profiles. We first emulated a dynamic exposure program resembling a typical profile of nutritional or pharmacokinetic stimulation (Figure 3C). Notably, the concentrations measured closely correlated with the theoretically derived concentrations in the device (Figure 3D). To assess whether the device could also simulate repeated exposure events, we next evaluated exposure patterns typical for daily serum insulin variations. Specifically, we introduced three peaks over the course of 24 h mimicking insulin elevations after breakfast, lunch and dinner (Figure 3E). The system reproducibly increased and decreased concentrations within physiologically relevant ranges with overall low variance (R^2^ = 0.87; Figure 3F).

Next, we aimed to evaluate the capability of the system to model chronic or repeated dosing. To this end, we conducted a 14‐day exposure experiment with repeated daily pharmacokinetic profiling using drug concentration measurements by mass spectrometry. Critically, we observed excellent alignment of the expected pharmacokinetic profiles and measured exposures without apparent drift over days or exposure cycles (Figure 3G). Daily pharmacokinetic profiles remained stable with reproducible c_max_, T_1/2_ and area under the exposure curves (AUC; Figures 3H–K). Combined, these results demonstrate that the pneumatically actuated device and sampling system can be automated to accurately mimic dynamic changes in exposure levels that are typical for translationally relevant physiological and pharmacological scenarios.

### 3.4 Emulation of APAP Hepatotoxicity Dynamics

**FIGURE 4:**
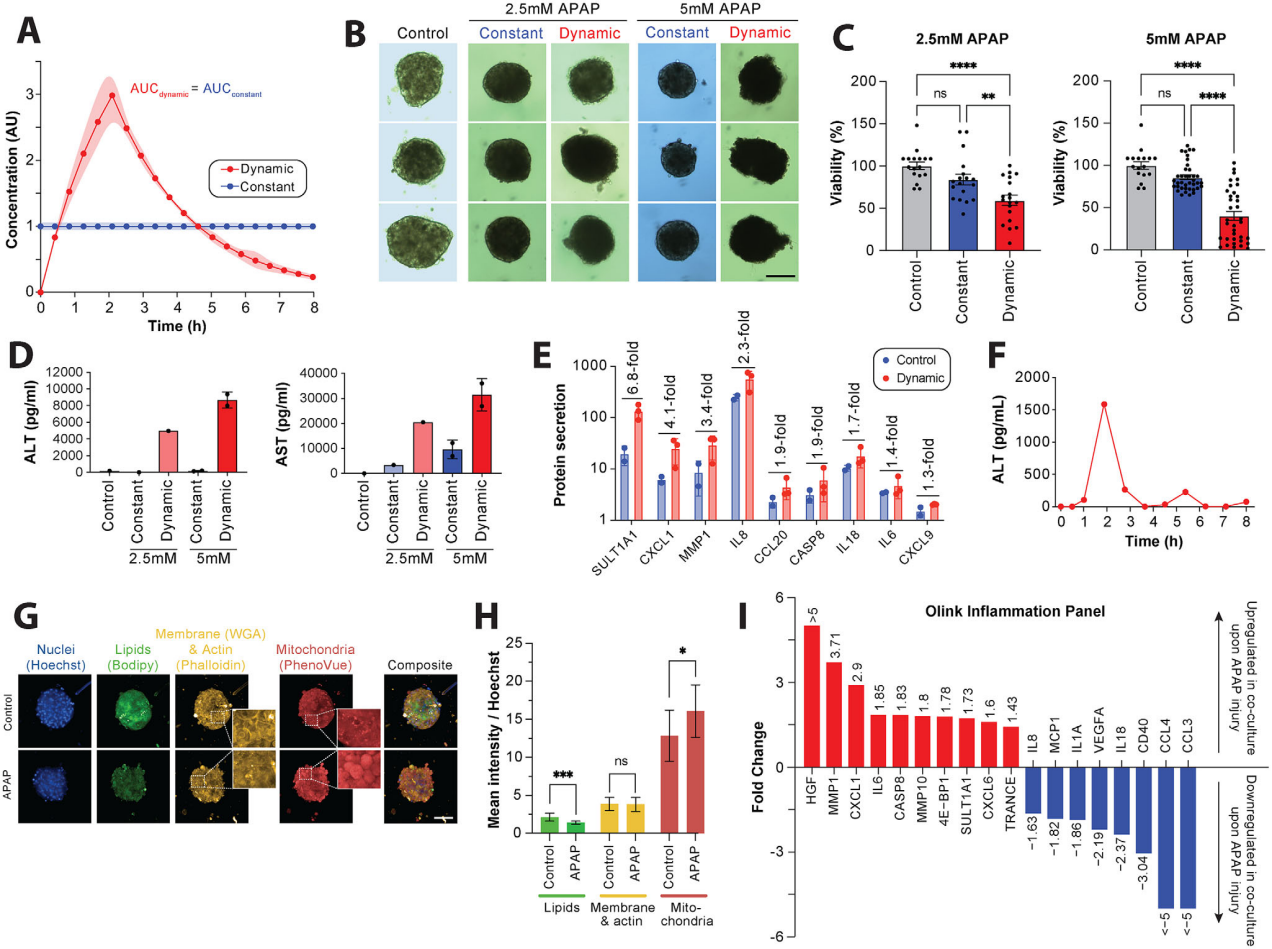
Exposure dynamics are critical to recapitulate acetaminophen hepatotoxicity. (A) Primary human liver spheroids were exposed on‐chip to different acetaminophen (APAP) exposure profiles—a constant exposure at a fixed concentration (blue; 2.5 mM or 5 mM) and a dynamic exposure mimicking the pharmacokinetic profile upon clinically manifest APAP overdose with equivalent area under the curve (AUC; red). The coefficient of variation was calculated for each timepoint, and relative variance bands are shown as upper and lower bounds (light blue and light red ribbons, respectively). (B) Brightfield images of three representative liver spheroids are shown after exposure to the different exposure profiles. (C) Cell viability (ATP levels normalized to controls) is shown after exposure to the different exposure regimens. Data are shown for 2.5 mM (left) and 5 mM (right) constant exposure and the AUC‐equivalent dynamic exposure pattern. *n* = 16–38 human liver spheroids per group. Error bars represent SEM. (D) Quantification of AST and ALT levels for the different exposure regimens. (E) Protein secretion profiles of bona fide markers of hepatocellular injury upon APAP overdose. Proteins are sorted by fold‐change. Error bars indicate SEM. (F) Representative quantification of ALT dynamics from sampled fractions. Note that the peak in ALT secretion is closely aligned with the peak in APAP exposure. (G) Cell Painting of human liver spheroids using multiplexed staining for nuclei (Hoechst33342; blue), lipids (BODIPY 493; green), actin and cell membranes (phalloidin & WGA; yellow) and mitochondria (PhenoVue‐641; red). Magnified inserts show collapse of actin cytoskeleton organization and diffuse release of mitochondrial dye in the phalloidin‐568 and PhenoVue‐641 channels, respectively. (H) Quantification of the signal intensity in panel G, normalized by the Hoechst signal. *n* = 14–19. Error bars indicate SEM. (I), Fold‐change in protein levels between PHH monoculture spheroids and PHH‐NPC co‐cultured spheroids exposed to the pharmacokinetic APAP regimen. Proteins in red and blue are upregulated and downregulated in co‐cultures versus monocultures, respectively. *N* = 3 independent experiments. *p < 0.05, **p < 0.01, ***p < 0.001 and ****p < 0.0001 in two‐tailed heteroscedastic t‐tests. ns indicates *p* > 0.05. Scale bars = 100 µm.

Next, we assessed whether exposure dynamics would impact cellular responses. To this end, we mimicked an acute APAP overdose scenario, which constitutes the most common cause of acute liver failure (ALF), accounting for around 40% of all cases globally [32, 33]. We cultured 3D human liver spheroids in the pneumatically actuated device. We compared a dynamic exposure profile to a constant exposure scenario with the same AUC_exposure_ (Figure 4A). This means that both scenarios used identical sampling and replenishment cycles and that cells in both regimens are exposed to the same number of APAP molecules; however, these are distributed differently over time. We tested two different APAP levels (2.5 and 5 mM); these correspond to exposures in severe APAP overdose patients, where plasma concentrations exceeding 3.5 mM have been reported [24], with first‐pass liver concentrations likely to be even higher. Upon microscopic inspection, spheroids exposed to the dynamic program were darker and exhibited a loss of clear spheroid borders, whereas spheroids exposed to the constant exposure appeared similar to controls (Figure 4B). This result was mirrored at the level of viability, where we observed that only the pharmacokinetically relevant exposure pattern resulted in a significant reduction in cellular ATP (Figure 4C).

To further characterize liver toxicity, we quantified alanine aminotransferase (ALT) and aspartate aminotransferase (AST) release as common clinical markers of liver injury (Figure 4D). AST levels showed dose‐dependent increases in both constant and pharmacokinetic exposure regimens; however, secretion levels were more than 3‐fold higher in the dynamic program. For ALT, elevations were exclusively detectable in the pharmacokinetically relevant program. Overall, we measured an AST:ALT ratio of approximately 4:1. In severe APAP poisoning in vivo, the formation of reactive metabolites can cause acute mitochondrial dysfunction, which results in the release of mitochondrial AST. Consequently, in overdose patients more severe hepatotoxicity is associated with AST/ALT ratios of ≥2:1 [34]. Besides ALT and AST, we observed significant increases in the secretion of proteins associated with cellular stress and injury, including IL6, IL8 and IL18, CASP8, CCL20 and CXCL1, as well as SULT1A1 (Figure 4E). Interestingly, when we analyzed release dynamics focusing on aminotransferase, we observed that ed after 2 h, closely aligned with c_max_ exposure (Figure 4F). Combined, these data demonstrate that our model can recapitulate APAP injury at relevant concentrations and timeframes. From a pharmacodynamic perspective, our results provide strong evidence that peak APAP concentration rather than cumulative dose is the central determinant of its hepatotoxicity.

To investigate the molecular mechanisms underlying the observed differences in hepatotoxicity, we first conducted multiplexed cytological profiling using Cell Painting (Figures 4G–H). We found that APAP reduced lipid levels, in agreement with previous reports of disrupted lipid homeostasis [35]. While actin levels did not change per se, the signal became more diffuse and did not clearly accumulate at membranes upon APAP toxicity, suggesting a loss of tight junctions and actin cytoskeleton detachment [36]. Staining for mitochondria indicated that the speckled pattern seen in control spheroids was replaced by a more diffuse pattern indicative of the release of mitochondrial contents into the cytosol. We furthermore conducted a quantitative multivariate analysis of the Cell Painting data based on a panel of cell and nuclear morphological features (Figure S1). The results show that cells become significantly hypertrophic while nuclei become less round and more elongated, indicative of a morphological injury phenotype. Nuclear shape is mechanically coupled to the actin cytoskeleton [37] and APAP‐associated disruption of the actin organization—as evidenced by more diffuse phalloidin staining—could thus deform nuclei and increase eccentricity. An increase in cell area may be linked to mitochondrial dysfunction and ATP depletion. When ATP levels drop, ion gradients and osmotic balance can fail, leading to cell swelling and, eventually, oncotic necrosis [38]. These findings thus demonstrate that multivariate morphological profiles can capture toxicity‐associated mechanistically interpretable phenotypes that align with independent biochemical markers of APAP‐induced injury.

Next, we evaluated the contribution of different hepatic cell types to APAP injury by comparing cytokine signatures between PHH monoculture spheroids and PHH‐NPC co‐cultures (Figure 4I). We used targeted proteomics to quantify 92 proteins using Olink proximity extension assay (PEA). Interestingly, we observed clear differences between the two culture setups. While CCL3, CCL4, IL8, IL18 and MCP1 were increased in monocultures, co‐culture spheroids showed a drastic increase in HGF, MMP1, MMP10, IL6 and CASP8 (Figure 4I). Elevation of CXCL1 suggests activation of pro‐inflammatory signaling and potential neutrophil recruitment, while increased CASP8 suggests the engagement of cell‐death‐associated signaling characteristic of APAP‐induced hepatotoxicity. These changes are consistent with the activation of Kupffer cells, cytokine‐mediated intercellular signaling, and stellate cell‐driven matrix remodeling. Concurrent increases in HGF and MMPs further indicate the induction of tissue remodeling and regenerative processes triggered by hepatic injury.

Combined, these results demonstrate that pharmacokinetically relevant exposure profiles can significantly affect cellular responses. These findings thus challenge the traditional reliance on static in vitro experiments for pharmacological and toxicological modeling and emphasize the importance of incorporating exposure dynamics into preclinical in vitro assessments.

## 4 Discussion

Over the past decade, microfluidic devices have been increasingly utilized for in vitro cell culture in dynamic environments [39]. While earlier studies typically cultured immortalized cell lines, the field has matured to the point that many current MPS also support the culture of primary human cells. These developments increase translational relevance and have led regulators to consider MPS data in regulatory applications, predominantly for safety assessments of small molecules [40]. However, there is also increasing interest in the use of MPS for the testing of antibody‐drug conjugates, antisense oligonucleotides (ASOs) and cell therapies [41, 42].

The ideal MPS should possess the following features: (1) The device should support the culture of human tissue models with adequate molecular phenotypes and functionality, ideally for multiple weeks. (2) The system should allow for precise control of flow rates and media composition. (3) It should be possible to monitor relevant endpoints longitudinally without the need to interrupt or terminate the culture. (4) The platform should not absorb drugs or other molecules of interest. (5) It is beneficial if the device is modular to allow for the integration and fluidic connection of multiple tissue models. In such cases, the system must allow appropriate medium conditioning in order to emulate tissue communication. (6) The platform should allow for the parallel operation of multiple devices. The current landscape of microfluidic in vitro systems is highly heterogeneous, and different platforms offer different advantages and limitations (Table 1). However, to the best of our knowledge, there are no current solutions that possess all of these features.

**TABLE 1:** Comparison of the presented solution with representative commercially available platforms.

Feature	This work	Emulate (S1 / R1)	CN Bio PhysioMimix	TissUse HUMIMIC	Mimetas OrganoPlate	InSphero Akura Flow	Draper PREDICT96	Dynamic42	Hesperos
**System architecture**	Closed recirculating	Single‐pass	Closed recirculating	Closed recirculating	Gravity bidirectional	Gravity bidirectional	Closed recirculating	Closed recirculating	Gravity unidirectional
**Device material**	OSTE+	S1: PDMS R1: Rigid PC	PDMS‐free	PDMS + PC + glass	PS + glass (PDMS‐free)	PS (PDMS‐free)	Thermoplastic	Proprietary polymer	PMMA
**Drug absorption**	Very low (up to 1500× < PDMS)	High (S1) Low (R1)	Very low	High risk	Low	Low	Low	Likely low	Low
**Perfusion**	Active instrument‐controlled perfusion	Active instrument‐controlled perfusion	Active instrument‐controlled perfusion	Active instrument‐controlled perfusion	Passive gravity‐driven perfusion	Passive gravity‐driven perfusion	Integrated micropump‐driven controlled perfusion	Active instrument‐controlled perfusion	Passive gravity‐driven perfusion
**Automated sampling**	Yes	Robotic liquid handler	No	Robotic liquid handler	Robotic liquid handler	No	Robot‐compatible	No	No
**Dynamic drug/ nutrient control**	Yes (programmable)	No	No	Yes (AutoLab)	No	No	Yes	No	Yes
**Automated media replenishment**	Yes	Yes	No	Yes (AutoLab)	Robot‐compatible	No	Yes	No	No
**Real‐time feedback control**	Yes (SegFormer machine vision)	No	No	No	No	No	No	No	No
**Real‐time monitoring**	Microscopy	Microscopy	No	Microscopy, O_2_, TEER	OrganoTEER	No	TEER, O_2_	TEER sensor, O_2_	MEA, cantilevers
**Max. culture duration demonstrated**	Weeks (demonstrated ≥2 weeks)	∼14 days	≥28 days	≥28 days	∼15 days	6–14 days	Weeks	Up to 7 days	28 days
**Throughput (per run)**	4‐12 chips per pressure controller	12–48 chips	48 wells per plate	Up to 24 chips	40–96 per plate	8–24 channels	96 per plate	2‐5 chips per pump	n/a

Abbreviations: MEA, microelectrode array; TEER, transepithelial electrical resistance.

The present study introduces a pneumatically actuated microfluidic platform that integrates automated longitudinal sampling, real‐time machine vision–based feedback control and programmable dynamic exposure regimens for the long‐term culture of 3D primary human liver microtissues. Previous seminal microfluidic in vitro platforms demonstrated that exposure to pharmacokinetically relevant exposure profiles can improve alignment with in vivo data and increase translational relevance [43, 44]. However, while technically elegant, these systems only used cancer cell lines for response profiling that do not closely mimic the phenotypes and functionality of human tissue. We and others previously demonstrated that liver spheroids cultured in the same media used here, exhibit mature hepatic phenotypes at the transcriptomic [45], proteomic [23] and metabolomic levels [46] for multiple weeks, resulting in superior sensitivity and improved predictive accuracy for preclinical assessments of drug metabolism [47, 48], drug‐drug interactions [49, 50] and toxicity [51, 52]. Furthermore, spheroids can accurately recapitulate liver regeneration [53, 54] and liver diseases, including metabolic dysfunction‐associated steatotic liver disease (MASLD), fibrosis and cholestasis [55, 56, 57, 58]. Thus, liver spheroids closely recapitulate human liver function for extended periods of time, rendering them well positioned for studies of human drug metabolism, liver toxicity, and disease.

Importantly, by integrating automated longitudinal sampling, real‐time machine vision–based feedback control and programmable dynamic exposure regimens, the solution presented here addresses the main limitations of closed‐loop systems, which include their incompatibility with continuous long‐term monitoring without manual intervention and their inability to replicate physiologically relevant pharmacokinetic, hormonal or nutritional dynamics. Specifically, our feedback‐controlled valve architecture maintains stable culture volumes over thousands of actuation cycles while enabling longitudinal low‐volume sampling. For instance, by sampling and replenishing 2 µl of the total 120 µl culture volume every 2 h, the system exchanges 20% of the medium per day, providing sufficient medium exchange to replenish nutrients and remove waste products without perturbing culture homeostasis. At the same time, extracted samples allow for longitudinal profiling of ultradian processes, which opens opportunities for continuous biomarker profiling, dynamic secretome analyses and real‐time monitoring of tissue responses—critical tools for investigative toxicology and systems pharmacology. The low variance of both extraction and replenishment volumes demonstrates the reliability of the fluidic control architecture and highlights its suitability for long‐term, high‐content studies. Furthermore, the close correlation between theoretical and measured concentration profiles underscores that the technical precision is sufficient to model dynamic human exposure regimens. Together, these advances enable a more faithful recreation of human tissue exposure dynamics and more accurate emulation of time‐dependent biological responses in mature organotypic human tissue models.

In parallel with advances in fluidic control and automation, substantial progress is being made toward increasing the biological complexity. Current major trends include co‐culture of different cell types in 3D organotypic cultures [59], the use of patient‐derived material [60, 61], integration of immune cells [62] and vascularization [63, 64]. These approaches are important for recreating tissue‐specific microenvironments and for emulating responses that depend on complex cell interactions, for example interactions between hepatocytes and stromal or endothelial cells. The platform presented here allows for the future integration of such 3D microtissues with increased complexity, which could further enhance the physiological relevance and translational utility of MPS models.

Throughput remains one of the critical limitations of MPS platforms, which restricts their use to specialized deep‐phenotyping studies rather than large‐scale pharmacological screening campaigns. From the perspective of fluidic architecture, the solution presented here can operate 4–12 chips per pressure controller in parallel, depending on the number of reservoirs and the complexity of the exposure program required for a given experiment. This level of multiplexing is comparable to that of other MPS platforms that rely on active, instrument‐controlled perfusion (Table 1). However, practical scalability is also constrained by non‐fluidic factors. In particular, each independently operated device currently requires dedicated machine‐vision feedback to monitor chamber volumes and adjust pneumatic actuation in real time. Thus, available computational resources, camera positioning and image‐analysis bandwidth can become limiting when multiple devices are run simultaneously. In addition, incubator space can restrict experimental scale, especially when devices, tubing, pressure lines, reservoirs and imaging components must all be accommodated under standard 37°C and 5% CO_2_ culture conditions. Several strategies can mitigate these limitations. Smaller custom‐made desktop incubators, in which temperature and CO_2_ levels are maintained locally, can increase operational flexibility and reduce dependence on conventional incubator space [65]. For wider dissemination and routine use, however, further miniaturization would be beneficial. In particular, replacing external camera‐based feedback with compact integrated sensors could substantially reduce the platform's footprint and facilitate higher degrees of parallelization while preserving the key advantage of closed‐loop fluidic control.

For translational pharmacological and toxicological assays, it is critical that the material of the culture platform does not interact with drugs or analytes of interest. The majority of MPS platforms are fabricated from PDMS, a porous elastomer widely known for its extensive absorption of small molecules into the microfluidic device bulk [66]. Here, we used OSTE as the platform material since it combines ease‐of‐fabrication with up to 1,500‐fold lower absorption compared to PDMS, particularly for hydrophobic compounds with a logP ≥2 [31]. By preserving free drug concentrations, this design improves the sensitivity of cultured liver spheroids, resulting in better prediction of liver safety. The top layers made of PDMS and PMMA never came in contact with the culture medium or the added drugs. Since tubing can also be a source of substantial absorption [67], we here used PTFE tubing precoated with a proteinaceous solution of 1% BSA. Together, these design and fabrication strategies contribute to the translational accuracy of the platform for preclinical drug testing, the importance of which will continue to grow given that the proportion of hydrophobic drugs in drug development pipelines has continuously increased over the past decades [68].

The utility of the model for toxicological applications is demonstrated by emulating the effects of acute APAP overdose. APAP hepatotoxicity depends on metabolic transformation by CYP2E1 and CYP3A4 to N‐acetyl‐*p*‐benzoquinonimine (NAPQI), a highly reactive metabolite responsible for APAP hepatotoxicity [69]. This means that the manifestation of APAP toxicity requires metabolically active hepatocytes and sensitivity to APAP is commonly included as a metric for evaluating hepatocyte functionality [70]. A multitude of studies have reported APAP toxicity in vitro; however, toxicity in these studies was either detected after prolonged exposure over multiple days [71, 72, 73, 74], which does not recapitulate the clinical course of APAP poisoning, or only at very high concentrations (often >10 mM), which exceeds overdose exposure levels [75, 76, 77]. Here, we show that APAP liver injury is detected at relevant concentrations and within relevant timeframes (2 h) when liver spheroids are exposed to patterns resembling in vivo APAP pharmacokinetics, as evidenced by elevated AST/ALT, increased cytokine production and reduced viability. In contrast, conventional exposure to the equivalent constant concentration with identical AUC_exposure_ did not elicit significant liver injury. These findings recapitulate clinical observations and demonstrate that APAP peak concentration rather than cumulative dose determines toxicity. The observed elevated AST:ALT ratio further mirrors patterns in overdose patients [34], emphasizing that peak‐dependent mitochondrial injury cannot be inferred from static dose–response experiments. These results provide direct experimental support for the concept that exposure dynamics rather than exposure magnitude per se are important determinants of toxicity that should be incorporated into preclinical toxicity testing frameworks.

Moreover, the proteomic signatures obtained from targeted Olink profiling highlight the platform's suitability for multimodal mechanistic studies. Increases in IL6, IL18, CASP8, CXCL1, and stress‐associated regulators corroborate known pathways of APAP‐induced injury, including sterile inflammation, apoptosis/necrosis, and compensatory regenerative signaling [78]. Comparisons between monoculture spheroids and PHH–NPC co‐cultures further indicate important contributions of non‐parenchymal cells to injury amplification, pro‐inflammatory cytokine release, and matrix remodeling. Our results contrast with a previous in vitro study that reported protective effects of NPCs [74], possibly because NPCs in this previous work were already activated at the time of seeding, which can result in repression of CYP activity and consequent reduction in NAPQI formation [79]. Instead, our results are consistent with in vivo findings that immune cell activation plays a critical role in APAP hepatotoxicity [80], further corroborating the translational benefits of pharmacokinetically relevant exposure profiles in organotypic human culture models.

In summary, the developed microfluidic platform with recirculating architecture and machine vision feedback control provides a robust, automated, and physiologically relevant solution for dynamic 3D primary human tissue culture. The system supports different exposure programs with ultradian or circadian periodicity, for instance, hormonal regulation, sequential drug dosing or real‐time monitoring of adaptive tissue responses under external perturbation. Using APAP hepatotoxicity as an example, we demonstrate that exposure dynamics can encode critical information that should be considered in addition to overall concentrations in investigative toxicology. The system thus represents an important step toward human‐centric, mechanistic in vitro models that more accurately predict clinical responses.

## Consent

No written consent has been obtained from the patients as there is no patient identifiable data included in this case report/series.

## Conflicts of Interest

Volker M. Lauschke is the CEO and shareholder of HepaPredict AB, as well as co‐founder and shareholder of Shanghai Hepo Biotechnology Ltd. Jibbe Keulen, Yi Zhong and Volker M. Lauschke are inventors on a related patent application. The other authors declare no conflicts of interest.

## Supporting information




**Supporting File 1**: advs76256‐sup‐0001‐FigureS1.pdf.

**Supporting File 2**: advs76256‐sup‐0002‐TableS1.docx.

**Supporting File 3**: advs76256‐sup‐0003‐Captions.docx.

## Data Availability

The data that support the findings of this study are available from the corresponding author upon reasonable request.

## References

[1] M. Schlander , K. Hernandez‐Villafuerte , C.‐Y. Cheng , J. Mestre‐Ferrandiz , and M. Baumann , “How Much Does It Cost to Research and Develop a New Drug? A Systematic Review and Assessment,” Pharmacoeconomics 39 (2021): 1243–1269, 10.1007/s40273-021-01065-y. 34368939 PMC8516790

[2] A. Schuhmacher , M. Hinder , E. Brief , O. Gassmann , and D. Hartl , “Benchmarking R&D success Rates of Leading Pharmaceutical Companies: An Empirical Analysis of FDA Approvals (2006–2022),” Drug Discovery Today 30 (2025): 104291, 10.1016/j.drudis.2025.104291. 39805539

[3] Y. Zhou , Y. Zhang , H. Xu , et al., “Dynamic Clinical Trial Success Rates for Drugs in the 21st Century,” Nature Communications 16 (2025): 9537, 10.1038/s41467-025-64552-2. PMC12572394 41162353

[4] A. Sertkaya , T. Beleche , A. Jessup , and B. D. Sommers , “Costs of Drug Development and Research and Development Intensity in the US, 2000‐2018,” JAMA Network Open 7 (2024): 2415445, 10.1001/jamanetworkopen.2024.15445. PMC11214120 38941099

[5] V. K. Singh and T. M. Seed , “How Necessary Are Animal Models for Modern Drug Discovery?,” Expert Opinion on Drug Discovery 16 (2021): 1391–1397, 10.1080/17460441.2021.1972255. 34455867

[6] D. G. Hackam and D. A. Redelmeier , “Translation of Research Evidence From Animals to Humans,” JAMA 296 (2006): 1731. 17032985 10.1001/jama.296.14.1731

[7] P. Perel , I. Roberts , E. Sena , et al., “Comparison of Treatment Effects Between Animal Experiments and Clinical Trials: Systematic Review,” BMJ 334 (2007): 197, 10.1136/bmj.39048.407928.BE. 17175568 PMC1781970

[8] P. J. K. van Meer , M. Kooijman , C. C. G. Wied , E. H. M. Moors , and H. Schellekens , “The Ability of Animal Studies to Detect Serious Post Marketing Adverse Events is Limited,” Regulatory Toxicology and Pharmacology 64 (2012): 345–349. 22982732 10.1016/j.yrtph.2012.09.002

[9] J. Bailey , M. Thew , and M. Balls , “An Analysis of the Use of Animal Models in Predicting Human Toxicology and Drug Safety,” Alternatives to Laboratory Animals 42 (2014): 181–199, 10.1177/026119291404200306. 25068930

[10] D. Cook , D. Brown , R. Alexander , et al., “Lessons Learned From the Fate of AstraZeneca's Drug Pipeline: A Five‐Dimensional Framework,” Nature Reviews Drug Discovery 13 (2014): 419–431, 10.1038/nrd4309. 24833294

[11] J. Kim , B.‐K. Koo , and J. A. Knoblich , “Human Organoids: Model Systems for Human Biology and Medicine,” Nature Reviews Molecular Cell Biology 21 (2020): 571–584, 10.1038/s41580-020-0259-3. 32636524 PMC7339799

[12] S. Youhanna , A. M. Kemas , L. Preiss , et al., “Organotypic and Microphysiological Human Tissue Models for Drug Discovery and Development—Current State‐of‐the‐Art and Future Perspectives,” Pharmacological Reviews 74 (2022): 141–206, 10.1124/pharmrev.120.000238. 35017176

[13] T. K. Baker , T. R. V. Vleet , P. K. Mahalingaiah , et al., “The Current Status and Use of Microphysiological Systems by the Pharmaceutical Industry: The International Consortium for Innovation and Quality Microphysiological Systems Affiliate Survey and Commentary,” Drug Metabolism and Disposition 52 (2024): 198, 10.1124/dmd.123.001510. 38123948

[14] S. A. Carratt , C. L. Z. de Zafra , E. Oziolor , et al., “An Industry Perspective on the FDA Modernization Act 2.0/3.0: Potential Next Steps for Sponsors to Reduce Animal Use in Drug Development,” Toxicological Sciences 203 (2024): 28–34, 10.1093/toxsci/kfae122. 39298459

[15] E. Y. Adashi , D. P. O'Mahony , and I. G. Cohen , “The FDA Modernization Act 2.0: Drug Testing in Animals is Rendered Optional,” American Journal of Medicine 136 (2023): 853–854, 10.1016/j.amjmed.2023.03.033. 37080328

[16] D. M. Stresser , A. K. Kopec , P. Hewitt , et al., “Towards In Vitro Models for Reducing or Replacing the Use of Animals in Drug Testing,” Nature Biomedical Engineering 8 (2024): 930–935, 10.1038/s41551-023-01154-7. 38151640

[17] A. W. Harrell , K. Reid , J. Vahle , et al., “Endeavours Made by Trade Associations, Pharmaceutical Companies and Regulators in the Replacement, Reduction and Refinement of Animal Experimentation in Safety Testing of Pharmaceuticals,” Regulatory Toxicology and Pharmacology 152 (2024): 105683, 10.1016/j.yrtph.2024.105683. 39117168

[18] D. E. Ingber , “Human Organs‐on‐Chips for Disease Modelling, Drug Development and Personalized Medicine,” Nature Reviews Genetics 23 (2022): 467–491, 10.1038/s41576-022-00466-9. PMC8951665 35338360

[19] Y. Huang , T. Liu , Q. Huang , and Y. Wang , “From Organ‐on‐a‐Chip to Human‐on‐a‐Chip: A Review of Research Progress and Latest Applications,” ACS Sensors 9 (2024): 3466–3488, 10.1021/acssensors.4c00004. 38991227

[20] R. Noguchi , H. Kubota , K. Yugi , et al., “The Selective Control of Glycolysis, Gluconeogenesis and Glycogenesis by Temporal Insulin Patterns,” Molecular Systems Biology 9 (2013): MSB201319, 10.1038/msb.2013.19. PMC4039368 23670537

[21] R. Zandi Shafagh , S. Youhanna , J. Keulen , et al., “Bioengineered Pancreas–Liver Crosstalk in a Microfluidic Coculture Chip Identifies Human Metabolic Response Signatures in Prediabetic Hyperglycemia,” Advanced Science 9 (2022): 2203368, 10.1002/advs.202203368. 36285680 PMC9731722

[22] E. Xie , W. Wang , Z. Yu , A. Anandkumar , J. M. Alvarez , and P. Luo , SegFormer: Simple and Efficient Design for Semantic Segmentation With Transformers, preprint, arXiv (2021), 10.48550/arXiv.2105.15203.

[23] C. C. Bell , D. F. G. Hendriks , S. M. L. Moro , et al., “Characterization of Primary Human Hepatocyte Spheroids as a Model System for Drug‐Induced Liver Injury, Liver Function and Disease,” Scientific Reports 6 (2016): 25187, 10.1038/srep25187. 27143246 PMC4855186

[24] L. F. Prescott , “Paracetamol (Acetaminophen) Poisoning: The Early Years,” British Journal of Clinical Pharmacology 90 (2024): 127–134, 10.1111/bcp.15903. 37683599

[25] D. M. Feidt , K. Klein , U. Hofmann , et al., “Profiling Induction of Cytochrome P450 Enzyme Activity by Statins Using a New Liquid Chromatography‐Tandem Mass Spectrometry Cocktail Assay in Human Hepatocytes,” Drug Metabolism and Disposition 38 (2010): 1589–1597, 10.1124/dmd.110.033886. 20551241

[26] C. Stringer and M. Pachitariu , “Cellpose3: One‐Click Image Restoration for Improved Cellular Segmentation,” Nature Methods 22 (2025): 592–599, 10.1038/s41592-025-02595-5. 39939718 PMC11903308

[27] F. Y. Zhou , Z. Marin , C. Yapp , et al., “Universal consensus 3D segmentation of cells From 2D segmented stacks,” Nature Methods 22 (2025): 2386–2399, 10.1038/s41592-025-02887-w. 41219412 PMC12615268

[28] A. E. Carpenter , T. R. Jones , M. R. Lamprecht , et al., “CellProfiler: Image Analysis Software for Identifying and Quantifying Cell Phenotypes,” Genome Biology 7 (2006): R100, 10.1186/gb-2006-7-10-r100. 17076895 PMC1794559

[29] O. Siméoni , H. V. Vo , M. Seitzer , et al., DINOv3, preprint, arXiv (2025), 10.48550/arXiv.2508.10104.

[30] N. Hollmann , S. Müller , L. Purucker , et al., “Accurate Predictions on Small Data With a Tabular Foundation Model,” Nature 637 (2025): 319–326, 10.1038/s41586-024-08328-6. 39780007 PMC11711098

[31] A. M. Kemas , R. Zandi Shafagh , N. Taebnia , et al., “Compound Absorption in Polymer Devices Impairs the Translatability of Preclinical Safety Assessments,” Advanced Healthcare Materials 13 (2024): 2303561, 10.1002/adhm.202303561. 38053301 PMC11469150

[32] G. Ostapowicz , R. J. Fontana , F. V. Schiødt , et al., “Results of a Prospective Study of Acute Liver Failure at 17 Tertiary Care Centers in the United States,” Annals of Internal Medicine 137 (2002): 947–954, 10.7326/0003-4819-137-12-200212170-00007. 12484709

[33] A. M. Larson , J. Polson , R. J. Fontana , et al., “Acetaminophen‐Induced Acute Liver Failure,” Hepatology 42 (2005): 1364–1372, 10.1002/hep.20948. 16317692

[34] R. M. Curtis and M. L. A. Sivilotti , “A Descriptive Analysis of Aspartate and Alanine Aminotransferase Rise and Fall Following Acetaminophen Overdose,” Clinical Toxicology 53 (2015): 849–855, 10.3109/15563650.2015.1077968. 26294195

[35] M. Suciu , A. T. Gruia , D. V. Nica , S. M. R. Azghadi , A. A. Mic , and F. A. Mic , “Acetaminophen‐Induced Liver Injury: Implications for Temporal Homeostasis of Lipid Metabolism and Eicosanoid Signaling Pathway,” Chemico‐Biological Interactions 242 (2015): 335–344, 10.1016/j.cbi.2015.10.019. 26522476

[36] W. Gamal , P. Treskes , K. Samuel , et al., “Low‐Dose Acetaminophen Induces Early Disruption of Cell–Cell Tight Junctions In Human Hepatic Cells And Mouse Liver,” Scientific Reports 7 (2017): 37541, 10.1038/srep37541. 28134251 PMC5278402

[37] S. Guixé‐Muntet , M. Ortega‐Ribera , C. Wang , et al., “Nuclear Deformation Mediates Liver Cell Mechanosensing in Cirrhosis,” JHEP Reports 2 (2020): 100145, 10.1016/j.jhepr.2020.100145. 32939447 PMC7479345

[38] H. Jaeschke , A. Ramachandran , X. Chao , and W.‐X. Ding , “Emerging and Established Modes of Cell Death During Acetaminophen‐Induced Liver Injury,” Archives of Toxicology 93 (2019): 3491–3502, 10.1007/s00204-019-02597-1. 31641808 PMC6891214

[39] H.‐R. Moon , N. Surianarayanan , T. Singh , and B. Han , “Microphysiological Systems as Reliable Drug Discovery and Evaluation Tools: Evolution From Innovation to Maturity,” Biomicrofluidics 17 (2023): 061504, 10.1063/5.0179444. 38162229 PMC10756708

[40] L. Ewart , E.‐M. Dehne , K. Fabre , et al., “Application of Microphysiological Systems to Enhance Safety Assessment in Drug Discovery,” Annual Review of Pharmacology and Toxicology 58 (2017): 65–82. 10.1146/annurev-pharmtox-010617-052722 29029591

[41] M. Mansouri , J. Lam , and K. E. Sung , “Progress in Developing Microphysiological Systems for Biological Product Assessment,” Lab on a Chip 24 (2024): 1293–1306, 10.1039/D3LC00876B. 38230512

[42] T. I. Maulana , N. R. Wevers , T. Kristoforus , et al., “Opportunities for Microphysiological Systems in Toxicity Testing of New Drug Modalities,” Annual Review of Pharmacology and Toxicology 65 (2025): 47–69, 10.1146/annurev-pharmtox-061724-080621. 39227343

[43] Y. A. Guerrero , D. Desai , C. Sullivan , et al., “A Microfluidic Perfusion Platform for In Vitro Analysis of Drug Pharmacokinetic‐Pharmacodynamic (PK‐PD) Relationships,” AAPS Journal 22 (2020): 53, 10.1208/s12248-020-0430-y. 32124093

[44] D. Singh , S. P. Deosarkar , E. Cadogan , et al., “A Microfluidic System That Replicates Pharmacokinetic (PK) Profiles In Vitro Improves Prediction of In Vivo Efficacy in Preclinical Models,” PLoS Biology 20 (2022): 3001624, 10.1371/journal.pbio.3001624. PMC9135222 35617197

[45] C. C. Bell , V. M. Lauschke , S. U. Vorrink , et al., “Transcriptional, Functional, and Mechanistic Comparisons of Stem Cell–Derived Hepatocytes, HepaRG Cells, and Three‐Dimensional Human Hepatocyte Spheroids as Predictive In Vitro Systems for Drug‐Induced Liver Injury,” Drug Metabolism and Disposition 45 (2017): 419–429, 10.1124/dmd.116.074369. 28137721 PMC5363699

[46] S. U. Vorrink , S. Ullah , S. Schmidt , et al., “Endogenous and Xenobiotic Metabolic Stability of Primary Human Hepatocytes in Long‐Term 3d Spheroid Cultures Revealed by a Combination of Targeted and Untargeted Metabolomics,” FASEB Journal 31 (2017): 2696–2708, 10.1096/fj.201601375R. 28264975 PMC5434660

[47] J. Riede , B. M. Wollmann , E. Molden , and M. Ingelman‐Sundberg , “Primary Human Hepatocyte Spheroids as an In Vitro Tool for Investigating Drug Compounds With Low Hepatic Clearance,” Drug Metabolism and Disposition 49 (2021): 501–508, 10.1124/dmd.120.000340. 34074732

[48] L. C. Preiss , K. Georgi , V. M. Lauschke , and C. Petersson , “Comparison of Human Long‐Term Liver Models for Clearance Prediction of Slowly Metabolized Compounds,” Drug Metabolism and Disposition 52 (2024): 539–547, 10.1124/dmd.123.001638. 38604730

[49] D. F. G. Hendriks , S. U. Vorrink , T. Smutny , et al., “Clinically Relevant Cytochrome P450 3A4 Induction Mechanisms and Drug Screening in Three‐Dimensional Spheroid Cultures of Primary Human Hepatocytes,” Clinical Pharmacology & Therapeutics 108 (2020): 844–855, 10.1002/cpt.1860. 32320483

[50] N. Oliva‐Vilarnau , S. U. Vorrink , F. A. Büttner , et al., “Comparative Analysis of YAP/TEAD Inhibitors in 2D and 3D Cultures of Primary Human Hepatocytes Reveals a Novel Non‐Canonical Mechanism of CYP Induction,” Biochemical Pharmacology 215 (2023): 115755, 10.1016/j.bcp.2023.115755. 37607620

[51] S. U. Vorrink , Y. Zhou , M. Ingelman‐Sundberg , and V. M. Lauschke , “Prediction of Drug‐Induced Hepatotoxicity Using Long‐Term Stable Primary Hepatic 3D Spheroid Cultures in Chemically Defined Conditions,” Toxicological Sciences 163 (2018): 655–665, 10.1093/toxsci/kfy058. 29590495 PMC5974779

[52] W. R. Proctor , A. J. Foster , J. Vogt , et al., “Utility of Spherical Human Liver Microtissues for Prediction of Clinical Drug‐Induced Liver Injury,” Archives of Toxicology 91 (2017): 2849–2863, 10.1007/s00204-017-2002-1. 28612260 PMC5515971

[53] N. Oliva‐Vilarnau , S. U. Vorrink , M. Ingelman‐Sundberg , and V. M. Lauschke , “A 3D Cell Culture Model Identifies Wnt/ β‐Catenin Mediated Inhibition of p53 as a Critical Step during Human Hepatocyte Regeneration,” Advanced Science 7 (2020): 2000248, 10.1002/advs.202000248. 32775153 PMC7404138

[54] N. Oliva‐Vilarnau , C. M. Beusch , P. Sabatier , et al., “Wnt/β‐Catenin and NFκB Signaling Synergize to Trigger Growth Factor‐Free Regeneration of Adult Primary Human Hepatocytes,” Hepatology 79 (2024): 1337–1351, 10.1097/HEP.0000000000000648. 37870288 PMC11095891

[55] D. F. G. Hendriks , L. F. Puigvert , S. Messner , W. Mortiz , and M. Ingelman‐Sundberg , “Hepatic 3D Spheroid Models for the Detection and Study of Compounds With Cholestatic Liability,” Scientific Reports 6 (2016): 35434, 10.1038/srep35434. 27759057 PMC5069690

[56] T. Hurrell , V. Kastrinou‐Lampou , A. Fardellas , et al., “Human Liver Spheroids as a Model to Study Aetiology and Treatment of Hepatic Fibrosis,” Cells 9 (2020): 964, 10.3390/cells9040964. 32295224 PMC7227007

[57] A. M. Kemas , S. Youhanna , R. Z. Shafagh , and V. M. Lauschke , “Insulin‐Dependent Glucose Consumption Dynamics in 3D Primary Human Liver Cultures Measured by a Sensitive and Specific Glucose Sensor With Nanoliter Input Volume,” FASEB Journal 35 (2021): 21305, 10.1096/fj.202001989RR. PMC12266331 33566368

[58] S. Youhanna , A. M. Kemas , S. C. Wright , et al., “Chemogenomic Screening in a Patient‐Derived 3D Fatty Liver Disease Model Reveals the CHRM1‐TRPM8 Axis as a Novel Module for Targeted Intervention,” Advanced Science 12 (2025): 2407572, 10.1002/advs.202407572. 39605182 PMC11744578

[59] A. Baze , C. Parmentier , D. F. G. Hendriks , et al., “Three‐Dimensional Spheroid Primary Human Hepatocytes in Monoculture and Coculture With Nonparenchymal Cells,” Tissue Engineering Part C: Methods 24 (2018): 534–545, 10.1089/ten.tec.2018.0134. 30101670

[60] S. Youhanna , N. Taebnia , Y. Liang , et al., “Primary Human Tissue Models for Metabolic Dysfunction‐Associated Liver Disease ‐ Toward Streamlining Drug Discovery With Patient‐Derived Assays,” Advanced Biology 9 (2025): 00337, 10.1002/adbi.202500337. PMC12712776 41200938

[61] B. J. Dwyer and J. E. E. Tirnitz‐Parker , “Patient‐Derived Organoid Models to Decode Liver Pathophysiology,” Trends in Endocrinology & Metabolism 36 (2025): 235–248, 10.1016/j.tem.2024.07.019. 39191607

[62] Y. Li , Y. Nie , X. Yang , et al., “Integration of Kupffer Cells Into Human IPSC‐Derived Liver Organoids for Modeling Liver Dysfunction in Sepsis,” Cell Reports 43 (2024): 113918, 10.1016/j.celrep.2024.113918. 38451817

[63] D. S. Veliz , K. Lin , and C. Sahlgren , “Organ‐on‐a‐Chip Technologies for Biomedical Research and Drug Development: A Focus on the Vasculature,” Smart Medicine 2 (2023): 20220030, 10.1002/SMMD.20220030. PMC7614466 37089706

[64] Y. Zhi , J. Wang , D. Huang , and Y. Zhao , “Self‐Organized Vascularized Hepatic Organoids in Microcapsules for Liver Regeneration,” Research 8 (2025): 0898, 10.34133/research.0898. 40979561 PMC12446754

[65] M. F. Wesseler , N. Taebnia , S. Harrison , et al., “3D Microperfusion of Mesoscale Human Microphysiological Liver Models Improves Functionality and Recapitulates Hepatic Zonation,” Acta Biomaterialia 171 (2023): 336–349, 10.1016/j.actbio.2023.09.022. 37734628

[66] M. W. Toepke and D. J. Beebe , “PDMS Absorption of Small Molecules and Consequences in Microfluidic Applications,” Lab on a Chip 6 (2006): 1484, 10.1039/b612140c. 17203151

[67] T. E. Winkler and A. Herland , “Sorption of Neuropsychopharmaca in Microfluidic Materials for In Vitro Studies,” ACS Applied Materials & Interfaces 13 (2021): 45161–45174, 10.1021/acsami.1c07639. 34528803 PMC8485331

[68] B. Faller , G. Ottaviani , P. Ertl , G. Berellini , and A. Collis , “Evolution of the Physicochemical Properties of Marketed Drugs: Can History Foretell the Future?,” Drug Discovery Today 16 (2011): 976–984, 10.1016/j.drudis.2011.07.003. 21782967

[69] E. M. Lancaster , J. R. Hiatt , and A. Zarrinpar , “Acetaminophen Hepatotoxicity: An Updated Review,” Archives of Toxicology 89 (2014): 193–199, 10.1007/s00204-014-1432-2. 25537186

[70] H. Jaeschke , O. B. Adelusi , J. Y. Akakpo , et al., “Recommendations for the Use of the Acetaminophen Hepatotoxicity Model for Mechanistic Studies and How to Avoid Common Pitfalls,” Acta Pharmaceutica Sinica B 11 (2021): 3740–3755, 10.1016/j.apsb.2021.09.023. 35024303 PMC8727921

[71] S. Messner , I. Agarkova , W. Moritz , and J. M. Kelm , “Multi‐Cell Type Human Liver Microtissues for Hepatotoxicity Testing,” Archives of Toxicology 87 (2013): 209–213, 10.1007/s00204-012-0968-2. 23143619 PMC3535351

[72] C. C. Bell , A. C. A. Dankers , V. M. Lauschke , et al., “Comparison of Hepatic 2D Sandwich Cultures and 3D Spheroids for Long‐Term Toxicity Applications: A Multicenter Study,” Toxicological Sciences 162 (2018): 655–666, 10.1093/toxsci/kfx289. 29329425 PMC5888952

[73] A. J. Foster , B. Chouhan , S. L. Regan , et al., “Integrated In Vitro Models for Hepatic Safety and Metabolism: Evaluation of a Human Liver‐Chip and Liver Spheroid,” Archives of Toxicology 93 (2019): 1021–1037, 10.1007/s00204-019-02427-4. 30915487

[74] C. C. Bell , B. Chouhan , L. C. Andersson , et al., “Functionality of Primary Hepatic Non‐Parenchymal Cells in a 3D Spheroid Model and Contribution to Acetaminophen Hepatotoxicity,” Archives of Toxicology 94 (2020): 1251–1263, 10.1007/s00204-020-02682-w. 32112222 PMC7225187

[75] S. J. Fey and K. Wrzesinski , “Determination of Drug Toxicity Using 3D Spheroids Constructed From an Immortal Human Hepatocyte Cell Line,” Toxicological Sciences 127 (2012): 403–411, 10.1093/toxsci/kfs122. 22454432 PMC3355318

[76] K. Takayama , K. Kawabata , Y. Nagamoto , et al., “3D Spheroid Culture of hESC/hiPSC‐Derived Hepatocyte‐Like Cells for Drug Toxicity Testing,” Biomaterials 34 (2013): 1781–1789, 10.1016/j.biomaterials.2012.11.029. 23228427

[77] Z. Wang , X. Luo , C. Anene‐Nzelu , et al., “HepaRG Culture in Tethered Spheroids as an In Vitro Three‐Dimensional Model for Drug Safety Screening,” Journal of Applied Toxicology 35 (2015): 909–917, 10.1002/jat.3090. 25512232

[78] M. R. McGill , “The Role of Mechanistic Biomarkers in Understanding Acetaminophen Hepatotoxicity in Humans,” Drug Metabolism and Disposition 52 (2024): 729–739, 10.1124/dmd.123.001281. 37918967 PMC11257692

[79] K. Klöditz , E. Tewolde , Å. Nordling , and M. Ingelman‐Sundberg , “Mechanistic, Functional, and Clinical Aspects of Pro‐inflammatory Cytokine Mediated Regulation of ADME Gene Expression in 3D Human Liver Spheroids,” Clinical Pharmacology & Therapeutics 114 (2023): 673. 37307233 10.1002/cpt.2969

[80] O. Krenkel , J. C. Mossanen , and F. Tacke , “Immune Mechanisms in Acetaminophen‐Induced Acute Liver Failure,” Hepatobiliary Surgery and Nutrition 3 (2014): 33143. 10.3978/j.issn.2304-3881.2014.11.01 PMC4273118 25568858

