# Supplementary figures and images for: A Machine Vision‐Guided Microphysiological Platform With Automated Microfluidics Enables Longitudinal Biomarker Monitoring and Emulation of Translationally Relevant Exposure Scenarios
Source: Adv Sci (Weinh). 2026 Jun 22:e76256. Online ahead of print. doi: 10.1002/advs.76256 (PMC13336620; doi:10.1002/advs.76256)

# Supplementary Figure 1

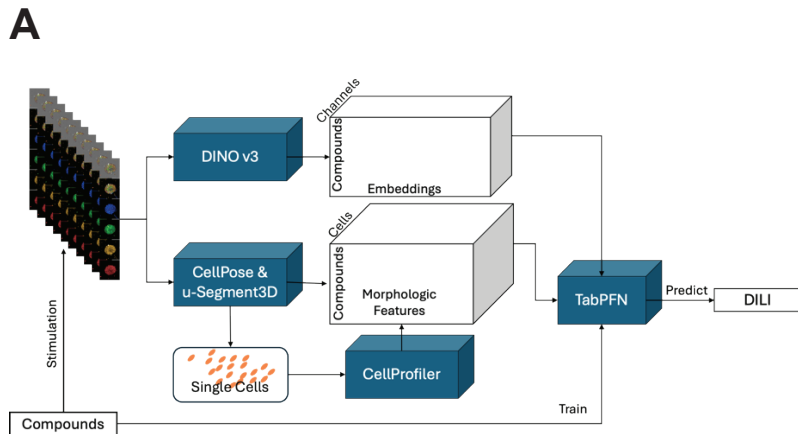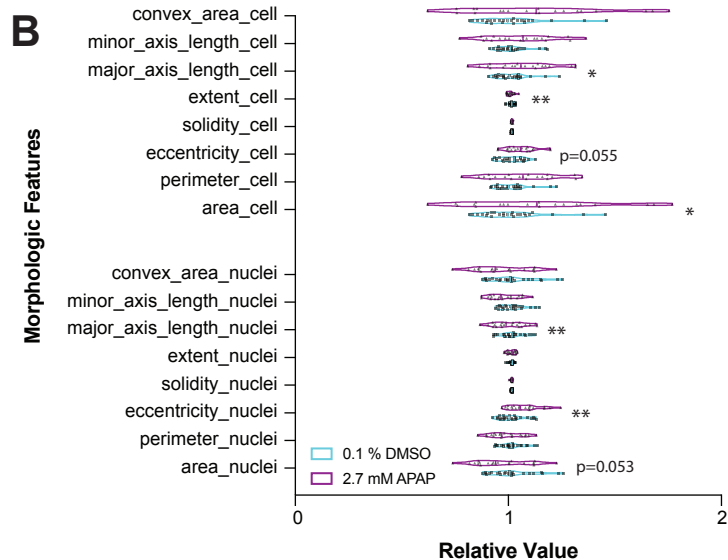

Supplement: Supplementary file 1 — Supporting File 1: advs76256‐sup‐0001‐FigureS1.pdf. [file ADVS-9999-e76256-s001.pdf]
