# Supplementary material for: A Machine Vision‐Guided Microphysiological Platform With Automated Microfluidics Enables Longitudinal Biomarker Monitoring and Emulation of Translationally Relevant Exposure Scenarios
Source: Adv Sci (Weinh). 2026 Jun 22:e76256. Online ahead of print. doi: 10.1002/advs.76256 (PMC13336620; doi:10.1002/advs.76256)
Supplement: Supplementary file 2 — Supporting File 2: advs76256‐sup‐0002‐TableS1.docx. [file ADVS-9999-e76256-s003.docx]

**Supplementary Table 1: Demographic and medical information of the liver cell donors used in this study.** BMI = Body mass index.

| **Donor** | **Sex** | **Age** | **BMI** | **Ethnicity** | **Medical history** |
| --- | --- | --- | --- | --- | --- |
| 1 | Female | 57 | 21.3 | Caucasian | Hypertension, on lisinopril, occasional alcohol intake |
| 2 | Female | 42 | 26.9 | Caucasian | No medication, no alcohol use |
| 3 | Female | 45 | 32.0 | Hispanic | No medication, social drinker |
