# Supplementary material for: A Machine Vision‐Guided Microphysiological Platform With Automated Microfluidics Enables Longitudinal Biomarker Monitoring and Emulation of Translationally Relevant Exposure Scenarios
Source: Adv Sci (Weinh). 2026 Jun 22:e76256. Online ahead of print. doi: 10.1002/advs.76256 (PMC13336620; doi:10.1002/advs.76256)
Supplement: Supplementary file 3 — Supporting File 3: advs76256‐sup‐0003‐Captions.docx. [file ADVS-9999-e76256-s002.docx]

**Supplementary Material**

Supplementary Tables: 1

Supplementary Figures: 1

**Supplementary Table 1: Demographic and medical information of the liver cell donors used in this study.** BMI = Body mass index.

**Supplementary Figure 1: 3D Cell Painting image-derived feature extraction workflow and morphologic readouts.** **A**, Multiplexed 3D confocal image stacks of compound-treated cells were processed with two complementary pipelines. Single-cell instances were first segmented using CellPose and u-Segment3D, and per-cell morphologic features were quantified with CellProfiler. In parallel, DINO v3 was applied to each channel of image volumes to learn self-supervised image embeddings. Morphologic features and embeddings were aggregated at the well level and used to train a TabPFN model to predict the drug-induced liver injury (DILI) label for each compound. **B**, Representative morphologic features for cytoplasm and nuclei illustrating the effect of 2.7 mM acetaminophen (APAP; magenta) relative to the vehicle control (DMSO; cyan). Each point represents the average value of an individual well, plotted as relative feature values normalized to the DMSO distribution. Selected features with differences approaching or reaching significance are annotated with p values, with asterisks denoting the corresponding significance level. * p<0.05 and ** p<0.01 in two-tailed heteroscedastic t-tests.
